# Reversible Disruption of Pre-Pulse Inhibition in Hypomorphic-Inducible and Reversible CB1^-/-^ Mice

**DOI:** 10.1371/journal.pone.0035013

**Published:** 2012-04-27

**Authors:** Maria Franca Marongiu, Daniela Poddie, Susanna Porcu, Maria Francesca Manchinu, Maria Paola Castelli, Valeria Sogos, Valentina Bini, Roberto Frau, Elisabetta Caredda, Maria Collu, Maria Serafina Ristaldi

**Affiliations:** 1 Institute of Genetic and Biomedical Research, National Research Council (IRGB-CNR), Monserrato, Italy; 2 Division of Neuroscience and Clinical Pharmacology, Department of Biomedical Sciences, University of Cagliari, Monserrato, Italy; 3 Center of Excellence for Neurobiology of Drug Dependence, University of Cagliari, Monserrato, Italy; Dulbecco Telethon Institute at San Raffaele Scientific Institute, Italy

## Abstract

Although several genes are implicated in the pathogenesis of schizophrenia, in animal models for such a severe mental illness only some aspects of the pathology can be represented (endophenotypes). Genetically modified mice are currently being used to obtain or characterize such endophenotypes. Since its cloning and characterization CB1 receptor has increasingly become of significant physiological, pharmacological and clinical interest. Recently, its involvement in schizophrenia has been reported. Among the different approaches employed, gene targeting permits to study the multiple roles of the endocannabinoid system using knockout (^-/-^) mice represent a powerful model but with some limitations due to compensation. To overcome such a limitation, we have generated an inducible and reversible tet-off dependent tissue-specific CB1^-/-^ mice where the CB1R is re-expressed exclusively in the forebrain at a hypomorphic level due to a mutation (IRh-CB1^-/-^) only in absence of doxycycline (Dox). In such mice, under Dox^+^ or vehicle, as well as in wild-type (WT) and CB1^-/-^, two endophenotypes motor activity (increased in animal models of schizophrenia) and pre-pulse inhibition (PPI) of startle reflex (disrupted in schizophrenia) were analyzed. Both CB1^-/-^ and IRh-CB1^-/-^ showed increased motor activity when compared to WT animals. The PPI response, unaltered in WT and CB1^-/-^ animals, was on the contrary highly and significantly disrupted only in Dox^+^ IRh-CB1^-/-^ mice. Such a response was easily reverted after either withdrawal from Dox or haloperidol treatment. This is the first Inducible and Reversible CB1^-/-^ mice model to be described in the literature. It is noteworthy that the PPI disruption is not present either in classical full CB1^-/-^ mice or following acute administration of rimonabant. Such a hypomorphic model may provide a new tool for additional in vivo and in vitro studies of the physiological and pathological roles of cannabinoid system in schizophrenia and in other psychiatric disorders.

## Introduction

Both genetic heterogeneity and clinical variability of schizophrenia, the complex mental disorder affecting as many as 51 million people worldwide, make it difficult to elucidate the neurobiological basis of clinical-association studies. Limited knowledge of the underlying molecular mechanism of such mental illness could be somewhat attributable to the difficulty in selecting experimental models that truly mimic schizophrenia [1]. Although several genes (including COMT, Dysbindin, Neuregulin, Synapsins, Arc, DISC etc.) have been implicated in its pathogenesis [2], a reliable animal model for such a severe mental illness is still lacking [3] and only some aspects of the pathology-endophenotypes- have been represented [4], [5].

Currently, genetically modified mice are being used to obtain or characterize such endophenotypes providing a molecular specificity impossible in human studies.

Since its cloning and characterization [6], CB1R has increasingly become of significant physiological, pharmacological and clinical interest. It represents the GPCR (G protein coupled receptor), most expressed in the central nervous system and in the periphery. The CB1R is an important component of the endocannabinoid system (eCBS) along with the CB2R, the endogenous cannabinoid ligands N-arachidonoylethanolamine or anandamide and 2-arachydonylglycerol, and the enzymes responsible for their synthesis and breakdown [7]. This system, and in particular CB1R, has generated considerable research interest both as a potential therapeutic target in the treatment of several pathological conditions, from neurological and psychiatric disorders to pain, metabolic syndromes and other diseases, as well as addiction [8].

The strong link between several mental illnesses and the cannabinoid system is widely documented [1]. CB1Rs are widely distributed in brain areas such prefrontal cortex, hippocampus and basal ganglia, all involved in schizophrenia In post-mortem studies of patients with schizophrenia a reduced CB1R mRNA and protein expression as well as altered CB1R density in prefrontal and cingulate cortices have been reported [9]. Moreover, several epidemiological studies have shown an increased frequency of cannabis abuse in schizophrenic patients (at least 50% of patients) [1], [3]–[5], [10] as well as an increased incidence of psychotic symptoms in people using it during adolescence [11]–[13]. A significant association between a human CNR1 gene polymorphism and a subtype of schizophrenia has been reported [14]–[15]. However, contradictory reports have been published on the association of CB1R gene polymorphism with schizophrenia and other authors have failed to identify a statistically significant segregation of CNR1 polymorphisms between schizophrenic patients and control groups [14]–[17].

Schizophrenic patients have symptoms that are considered positive (hallucination, delusions, disordered thinking and paranoia) and negative (deficit in social interaction, emotional expression and motivation). All the studies in animals, although providing reliable data, suffer from the difficulty in selecting an experimental model that resembles schizophrenia [1]. Moreover, only in few papers the CB1R as well as the eCBS system have been extensively examined in experimental (animal) models of schizophrenia, and even less in mouse models. In rats all models were based on the injection of PCP, a NMDA antagonist that induces psychotic symptoms in humans. In these experiments the dysregulation of CB1R in different brain areas is evident, but the extent of its alteration, as well as the brain areas involved, depends on the adopted model [1]. The few experiments carried out on CB1 KO mice also were done using PCP injections. They have shown that the CB1 gene disruption alters the behavioral effects of PCP with a reduction of locomotion (increased in WTs) and enhanced ataxia and stereotypy more than in controls, without affecting social interaction [18]. Based on these results it has been suggested that eCBS play different roles in negative and positive symptoms, facilitating the formers and inhibiting the latters. Furthermore, pharmacological studies, mostly on rats, indicate that CB1R agonists can either reduce or have no effect on positive symptoms induced by dopaminomimetic agents. On the contrary, a non psychoactive component of *C. Sativa as cannabidiol* dose-dependently inhibits the hyperlocomotion induced in mice by ketamine [19]. Therefore, an adaptive/protective role of eCBS in schizophrenia has been proposed [20]. Removing of eCBS control through either the pharmacological blockade of CB1R or, similarly, inducing its sensitization/internalization should enhance the positive symptoms by reducing the inhibitory activity of eCBS on dopaminergic pathways, thus worsening the symptoms [1]. Independently from experiments carried out using animal models of schizophrenia, it must be underlined that an endocannabinoid tone in the regulation of locomotor activity has been suggested by the stimulating effect of the antagonist SR141716A per se and by its ability in potentiating the locomotor stimulant effects of amphetamine and apomorphine [21]. This hypothesis is supported by the observation that locomotor activity is slightly increased in mice without cannabinoid receptors [21]. However, another study did not replicate this result and, on the contrary, a decrease in open-field activity in the Zimmer’s CB1 knockout strain was found [22]. These divergences have been explained by the biphasic effects of cannabinoids depending on the level of the endogenous tone, or by the observation that CB1 KO mice apparently have higher levels of anxiety. The results may have been inuenced by the experimental conditions (different intensity in illumination of the open-field), low light condition being less anxiogenic for mice that then exhibit higher locomotor activity [21].

Studies of sensorimotor gating of startle responses to strong exteroceptive stimuli provide an excellent method for exploring information processing and attentional deficits in schizophrenia. Prepulse inhibition (PPI) is defined as the decrease in the acoustic startle response when a non-startling prepulse is presented before the startling pulse [23]. Chronic schizophrenic and non-medicated first-episode schizophrenic patients have a marked deficit in PPI. In rodents, as well as in human health volunteers, such deficits can be obtained acting on different neuronal systems through stimulation of the dopaminergic D2 receptors (with amphetamine or apomorphine), activation of serotoninergic systems by direct 5-HT2A receptor agonists (with LSD or psilocybin) and blockade of NMDA receptors by drugs such as ketamine and PCP. Several studies show that the CB1agonists may alter PPI however with not always concordant results as CP55940 and WIN 55,212–2 reduced sensorimotor gating in rats [24]–[25] but no changes in PPI after WIN 55,212–2 treatment, at whatever dosage and schedule were observed in a different paper [26]. More recently it has been reported in mice [27] that THC impaired PPI, while also reducing the startle response. Moreover, the pretreatment with typical (haloperidol) and atypical (risperidone) antipsychotics, two potent dopamine D2 receptor antagonists, as well as the CB1R antagonist SR141716 reversed the THC-induced PPI deficit. However, in other studies in rats such ability of SR141716 to revert the PPI deficits induced by dopaminergic stimulation as well as the hyperlocomotion and stereotypy has been questioned suggesting that blockade of the CB1 receptor on its own is not sufficient for antipsychotic therapy [1]. Furthermore, one more study on rats reported that SR141716 behaves as an atypical antipsychotic since, similarly to clozapine, it counteracted the PPI disruption produced by the NMDA antagonists PCP and dizocilpine, and by apomorphine [28]. Finally, it has been demonstrated [29] that SR141716 antagonized the disruptive PPI effects of apomorphine also in mice. Moreover, Long and coworkers [30] showed that in mice cannabidiol (CBD) significantly reversed PPI deficits induced by MK-801 whereas did not affect PPI on its own. Because clozapine (4 mg/kg) gave the same results, it was concluded that CBD may have atypical antipsychotic potential. Thus, eCBS are probably implicated in the negative regulation of dopamine release by acting retrogradely on CB1 receptors in dopaminergic presynaptic terminals lowering dopamine release [1].

Among the schizophrenia endophenotypes, the pre-pulse inhibition (PPI) of the startle response is one of the most studied and has been described in different transgenic mice [31]. Moreover, PPI experiments in CB1 knockout (^-/-^) mice [32]–[35] have never been performed, although a very recent paper reporting the involvement of the CB2 receptor in such a phenomenon has been published [10]. The aim of our study was to investigate mainly the PPI response both in the conventional CB1^-/-^ mice and in our temporal inducible-reversible and tissue-specific hypomorphic model (IRh-CB1^-/-^) in which the CaMKIIα (Ca^2+^/calmodulin-dependent kinase IIα) promoter [35] modulates CB1R gene expression in specific brain areas. In these transgenic mice, CB1 knockdown is achieved through the Tetracycline-controlled transactivator (tTA) system and when Dox is present, transcription is off [36]–[38]. In this system, the tTA (deriving from the tetracycline resistance operon of *E. Coli* Tn10) can be engineered under the control of tissue-specific or ubiquitous promoters, while tetOpmin regulates the expression of a cDNA cloned in *cis*, i.e., the gene of interest (when Dox is present, transcription is off).

## Materials and Methods

### Animals

FVB/N, C75BL/10 and CBA/J (Harlan Europe, Italy) used to generate the transgenic line pBI-G-CB1 (see below) tTA-CaMKIIα (originally bred on CD1 background mice were kindly donated by Dr. Dan J Dumont, Molecular and Cellular Biology, Sunnybrook & Women’s, Toronto) and CB1^-/-^ mice (originally bred on C57BL/6J background were kindly donated by Dr. Aaron H. Lichtman, Department of Pharmacology & Toxicology, Virginia Commonwealth University) for behavioral experiments were group-housed (2–3 animals per cage) in colony cages (Litter Plus, SAFE, France) and maintained under standard conditions (temperature of 22±2°C, humidity 50%±1) with a 12-h light- dark cycle (lights on 07∶00 am) with food (A04 feed, SAFE, France) and water available ad libitum. All mutant lines were bred for >5 generations on the background of CBA/J-C57BL/10. CaMKII-tTA^+/-^ and pBI-G-CB1^+/-^ littermates (originating after backcrossing the different genotypes in CBA/J-C57BL/10) were used as wild-type (WT) for the behavioral and molecular experiments.

All procedures and experiments were carried out in an animal facility according to Italian (D.L. 116/92 and 152/06) and European Council directives (609/86 and 63/2010) and in compliance with the approved animal policies by the Ethical Committee for Animal Experiments (CESA, University of Cagliari) and the Italian Department of Health.

### Constructs

RNA samples extracted from the brain of adult mouse FVB/N (Trizol reagent Invitrogen cat.n°15596–018) were retrotranscribed by the SuperScript™ First-Strand Synthesis System for RT-PCR using oligo (dT) as a primer (Invitrogen Cat. N° 12371–019). A 1,495 Kb cDNA (from position –46 to position+1449) fragment encoding CB1 receptor was obtained by PCR reaction using primers specific for the CB1. The oligonucleotides have been designed with linkers containing consensus sequences for the restriction enzymes PstI and Sal1 (forward PstI-CB1 CTACTGCAGCTCTTTCTCAGTCACGTTG; reverse SalI-CB1 TATGTCGACTTCTGGGCAGCCACAAAAG). The Pst1 and Sal 1 restriction sites allow the in-frame directional cloning of the CB1 cDNA into the vector pBI-G Tet Vector (Clontech cat 6150-1 Gene Bank accession number U89933) (called pBI-G-CB1). In the pBI-G vector, the tetO operator controls two CMV promoters in opposite direction. One of the two promoters directs the CB1 cDNA, the other controls a reporter gene (*Lac Z*), allowing the temporal and spatial expression of the transgene. The final pBI-G-CB1 construct was mapped using restriction enzymes and fully sequenced. The cDNA construct was linearized by restriction enzyme digestion (BglII) and purified by phenol and chloroform extraction and precipitated with ethanol. The DNA was resuspended in Low Salt Buffer (0,2M NaCl, 20 mM TrisHCl, 1mM EDTA, pH 7.4) and filtered using an Elutip-D syringe column (Whatman) and diluted in 1 to 4 ng/µl in microinjection buffer (5 to 10 mM Tris pH 7.4, 0.1 to 0.25 mM EDTA). Purified DNA fragments were injected into fertilized mouse eggs (FVB/N) and then transferred into pseudo-pregnant foster mothers (C57/BL6). Founder animals were identified by PCR using primers specific for the *Lac Z* (forward GGCGTTACCCAACTTAATC, reverse ATGTGAGCGAGTAACAACC) and Southern blot (probe: PCR product of the *Lac Z* and the total plasmid used for microinjection). F1 progeny was obtained by breeding founder animals with the C57/BL6 mice. The transgenic line pBI-G-CB1 was crossed with the transgenic line for the tTA under the control of the tissue-specific promoter CaMKIIα and with emizygous CB1^+/–^.

The screening of the progeny was carried out by PCR on DNA from tail biopsy using three sets of primers. One specific for the pBI-G vector (*Lac Z*), one for the tTA transgene (forward: TTGATCACCAAGGTGCAG; reverse CTGCTCAAACTCGAAGTC) and the other for the Neo gene (CB1^-/-^ forward ATGGGATCGGCCATTGAAC; reverse CTCGTCCTGCAGTTCATTC). Animals emizygous for the three transgenes (pBI-G-CB1^+/–^; CaMKII tTA^+/–^ and CB1^+/–^) were mated to obtain mice homozygous for the CB1^-/-^ and emizygous for the pBI-G-CB1 (^+/–^) and homozygous/emizygous for CaMKII-tTA (^++;+/–^) (named Inducible Reversible CB1^-/-^ or IRh-CB1^-/-^). Screening was performed by PCR utilizing the following primers: forward CAATTTGTGGTGCCTGGTG and reverse TGGATGTTGTCCTCGTTC.

This murine model re-expresses the CB1R only in the brain areas where the CaMKII promoter is active (mainly the forebrain) and knocks it down anytime by adding the tetracycline analog Dox to the diet. The spatial and temporal expression of the transgene was determined by following the expression of the reporter gene *Lac Z* and the CB1 expression level by real-time PCR in animals treated with Dox versus untreated animals and WT.

### *Lac Z* Expression

Expression of *Lac Z* in the final transgenic line IR- CB1^-/-^ was assayed by staining brain slides for B galactosidase (*Lac Z*) activity [39]. Briefly, animals were sacrificed by cervical dislocation, brains quickly removed and frozen on dry ice. Frozen sections (20μm) were cut on a cryostat and fixed in 4% paraformaldehyde (PFA) in phosphate buffered saline (PBS) for 15 min, incubated in X-gal solution at room temperature overnight, and then washed with PBS. X-gal solution had the following composition: 0.5 mM potassium ferricyanide, 0.5mM potassium ferrocyanide, 2mM MgCl_2_, 0.1% Triton-X-100 and 0.037% X-gal.

### Real-time PCR

RNA samples were extracted from the prefrontal cortex, caudate nucleus, hippocampus and cerebellum of adult mice (IRh-CB1^-/-^) after 15 days of Dox treatment, without any treatment and WT used as controls. They were retrotranscribed by using the SuperScript™ First-Strand Synthesis System for RT-PCR using random primer (Invitrogen Cat. n° 12371–019). cDNAs were analyzed by quantitative real-time polymerase chain reaction (Q-RT PCR) with specific primers for the genes (Mn 00432621_S1 cnr1 Applied Biosystem). Q-RT PCR reactions were performed using the ABI Prism 7900 Fast Real Time PCR (Applied Biosystem) with TaqMan (TaqMan PCR 2X Master mix; Applied Biosystems).

### Immunohistochemistry

Immunochemistry experiments were performed as previously described [40]. Briefly, mice were anaesthetized with chloral hydrate (400 mg/kg, i.p.), and transcardially perfused with 4% paraformaldehyde and 0.1% glutaraldehyde in 0.1M phosphate-buffered saline (PBS, pH 7.4). After repeated washing in 0.1 M PBS, coronal sections (40 μm thick) of brain were prepared with a vibratome and immunostaining was performed on free-floating sections. Pre-blocking of tissue sections was performed with 10% normal goat serum, 2.5% bovine serum albumin (BSA) and 0.2% Triton X-100 in PBS for 1h at room temperature. Sections were incubated for 48 h at 4°C with rabbit anti-CB_1_ receptor polyclonal antibodies directed against the last 15 amino acids of rat CB_1_ receptor (1∶2000) [41] kindly supplied by Dr. K. Mackie (Department of Psychological & Brain Sciences and the Gill Center, Indiana University). After washing in PBS-0.2% Triton X-100, sections were incubated for 1h at room temperature with biotinylated goat anti-rabbit IgG (1∶200, Vector Laboratories, Burlingame, CA, USA). Subsequently, sections were incubated with Avidin Alexa Fluor^R^ 488 for 1 h in the dark at room temperature, then rinsed and mounted onto Superfrost glass slides in antifading solution with 200 mg/ml of 4′,6-diamidino-2-phenylindole (DAPI) as a nuclear counterstain. Standard control experiments were performed by omission of either the primary or secondary antibody and yielded no cellular labeling.

All observations were made using an Olympus IX 71 microscope (Olympus, Tokyo, Japan) equipped with 10×, 20× and 60× plan apochromatic oil immersion objectives (Olympus UPlanSApo series) with an efficient chromatic correction to minimize the focus drift between different fluorescence emissions. Images were taken with a 12-bit cooled CCD camera (Sensicam PCO, Kelheim, Germany) electronically coupled to a mechanical shutter interposed between the 100-W Hg lamp and the microscope, to avoid photo bleaching. The digital resolution of images made with the 60× objective was 0.1μm/pixel. Excitation light was attenuated with a 6% transmittance neutral density filter. Color compositions were made using images of antibodies as single RGB channels. When the immunosignal was small, as for the CB1R, the focus depth was extended by summing the maximum intensity of several images taken at focus steps of 1 μm. Image analysis and measurements were performed using the ImagePro Plus package (Media Cybernetics, Silver Springs, MD, USA).

### Behavioral Experiments

A total number of 87 (50 litters) male mice were used for this purpose. All mice were 2 to 4 months old and weighed from 25 to 40 g at the beginning of the experiments. All behavioral experiments were conducted between 11∶00 AM and 3∶00 PM in sound proofed rooms. For some experiments the same animals were used in each paradigm, and between each experimental paradigm mice were allowed to rest at least for 1 month. Locomotor activity was assessed as the first behavioral test.

To exclude possible aspecific effects of Dox treatment and/or influences of sucrose (energy intake) on behavioral experiments some mice were given either the antibiotic (n = 3) or sucrose (5%) in their water supply (n = 4). Since no differences were observed in both locomotor activity and PPI response, all of them have been included in the analysis of the data.

### Spontaneous Locomotor Activity

Mice were individually tested for motor activity under standardized environmental conditions (light intensity with a Digiscan Animal Activity Analyzer (Omnitech Electronics, Columbus, Ohio). Each cage (42 cm×30 cm×30 cm) had two sets of 16 photocells located at right angles to each other, projecting horizontal infrared beams 2.5 cm apart and 2 cm above the cage floor and a further set of 16 horizontal beams whose height could be adapted to the size of the animals (for the mice 12 cm above the cage floor). Basal horizontal and vertical activities were measured as total number of sequential infrared beam breaks in the horizontal or vertical sensors, recorded every 10 minutes, beginning immediately after placing the animals in the cage, over a period of 90 minutes. The output data are expressed as distance moved in cm (horizontal activity) and as number of rearing episodes (for vertical activity), and as time (sec) spent in the center of the arena.

### Acoustic startle Response and Pre-pulse Inhibition (PPI)

Detection of acoustic startle response and PPI was performed as described [42], in four standard non-restrictive Plexiglas cylinder cages (3.2 cm diameter × 12 cm length diameter) mounted on a piezoelectric accelerometer platform, all in sound-attenuated chambers with fan ventilation, connected to analogue–digital converters (Med Associates, St. Albans, VT, USA) with a 60-dB ambient noise level. Background noise and acoustic startle were conveyed through two speakers placed in proximity to the startle cage so as to produce a variation in sound intensity within 1 dB. On the testing day, each mouse was placed in a cage for a 5-min acclimatization period with a 70-dB white noise background, which continued for the remainder of the session. Each session consisted of three consecutive sequences of trials (periods). Unlike the first and the third periods, during which mice were presented with only five pulse-alone trials of 115 dB, the second period consisted of a pseudorandom sequence of 50 trials, including 12 pulse-alone trials, 30 trials of pulse preceded by 73-, 76-, or 82-dB pre-pulses (ten for each level of prepulse loudness), and eight no-stimulus trials, where only the background noise was presented. The duration of pulses and pre-pulses was 80 and 40 ms, respectively. Prepulse–pulse delay amounted to 100 ms. Inter-trial intervals were selected randomly between 10 and 15 s. Startle amplitude values were calculated as the difference between peak-to-peak voltage during a time window of 80 ms after stimulus onset and peak-to-peak voltage in the 80 ms time window before stimulus onset.

The percent (%) PPI was calculated based only on the values relative to the second block, and using the following formula: (100–((mean startle amplitude for prepulse+pulse trials/mean startle amplitude for pulse-alone trials)×100).

### Drugs and Treatments

Doxycycline HCl (SIGMA-ALDRICH, Italy) was dissolved in sterile 5% sucrose solution and administered to mice in sterilized bottles that were light protected by wrapping them with a black adhesive paper [43]. The *ad libitum* solution (50 µg/ml) was freshly prepared every 4 days, the treatment lasting 12 to 15 days. Haloperidol (Haldol®, Janssen - Cilag, Italy) dissolved in saline was administered intraperitonelly (i.p.) 30 min before PPI at 1 mg/kg/10 ml.

### Data Analysis

Results are expressed as the mean±SEM of *n* mice as specified in the text and/or figures legend. For the PPI test, the data were analysed as the average percent (%) PPI over the 3 prepulse intensities tested. The significance of differences between groups was determined by one-way or two-way analysis of variance (ANOVA or Kruskall-Wallis) followed by Newman–Keuls or Dunn’s Multiple Comparison or Bonferroni *post-hoc* comparisons when appropriate, or Student’s t test. The threshold for significant difference was set at *p*<0.05. Graphs and statistics were generated by Graph Pad Prism 5.0 (Graph Pad Software; http://www.graphpad.com).

## Results

### Generation and Characterization of IRh-CB1^-/-^ Mice

**Figure 1:**
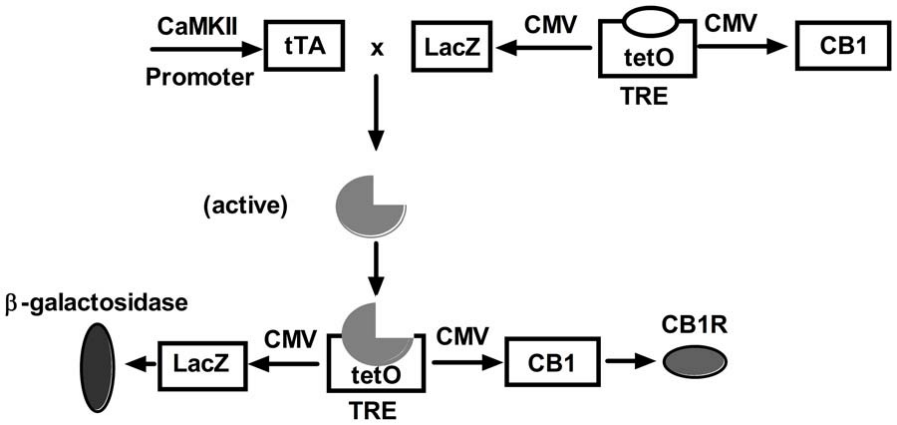
Schematic representation of the construct for generating the IR-CB1-/-. Tetracycline-controlled transactivator (tTA) expression was driven by the CaMKII promoter. In its active form, tTA binds to the tetO operator sequence that controls two promoters in opposite direction and initiates expression of the CB1 and Lac Z transgenes. Expression can be inhibited by Dox as it binds to tTA, making it inactive.

In Fig. 1, a schematic representation of the IRh-CB1^-/-^ model is shown. Six transgenic mice were produced, three of which did not express the transgene after crossing with the CaMKIIα–tTA transgenic line. From the remaining three lines, we used the mice with CB1R mRNA levels closest to physiological ones to carry out the crosses to generate IRh-CB1^-/-^. Animals with the following genotypes were used for all experiments: CB1^-/-^, PBI G/CB1^+/–^; tTA^+/–^; CB1^-/-^ (IRh-CB1^-/-^), and their littermates CaMKII-tTA^+/–^ and/or pBI-G-CB1^+/–^ as WT.

RT-PCR from total mRNA of brain IRh-CB1^-/-^ confirmed the production of a full-length CB1 RNA from the transgene (data not shown).

**Figure 2:**
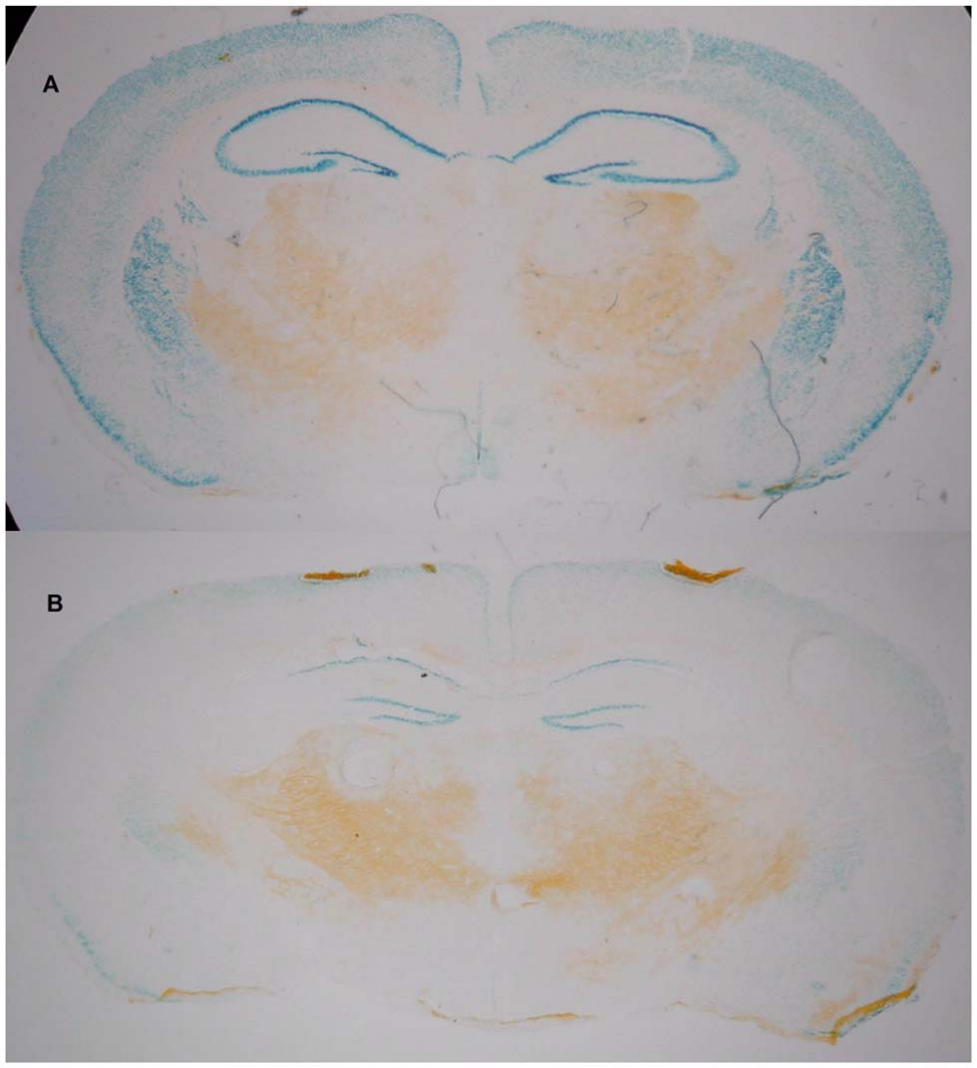
Sample of B-GAL activity in both WT and IR-hCB1^-/-^ mice either Dox treated or untreated. The expression of the reporter gene LacZ is visualized by β-GAL activity in the hippocampus from untreated (A) and Dox-treated (B) mice.

**Figure 3:**
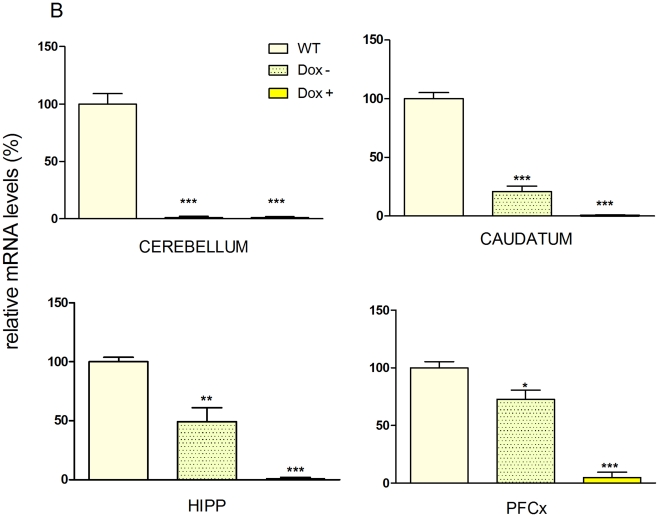
Real time-PCR CB1 mRNA expression in both WT and IR-hCB1^-/-^ mice either Dox treated or untreated. The mRNA expression detected by real time-PCR was measured in different brain areas from WT, untreated and Dox-treated IR-hCB1^-/-^ mice (n = 3 for each group). *P<0.001.

**Figure 4:**
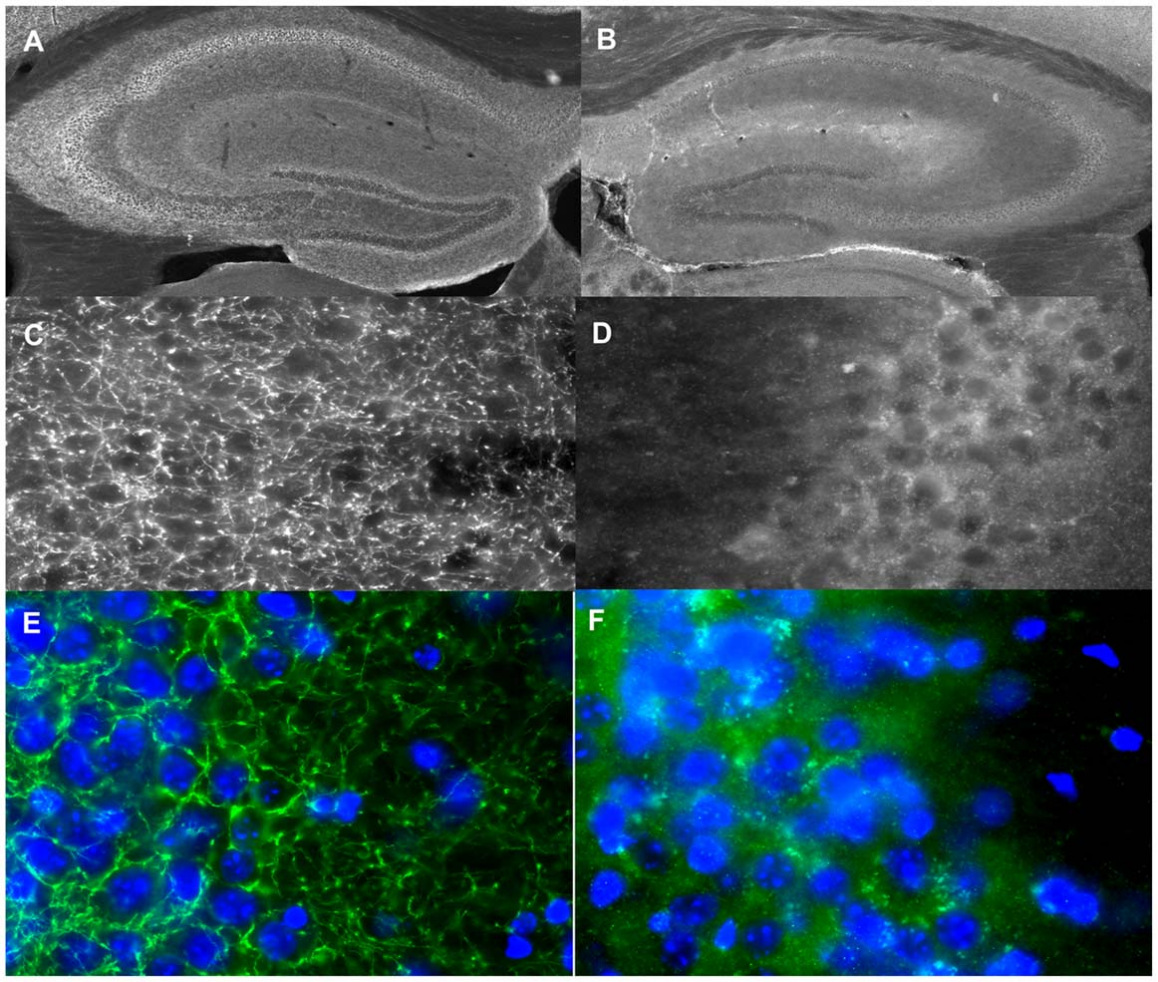
CB_1_ receptor immunoreactivity in the hippocampal formation. Representative low-magnification (4 x) showing CB1-IR throughout the hippocampus of WT (A) and IR-hCB1^-/-^ (B) mice. Higher-magnification (20 x) of the CG3 displays a dense network of CB1 immunoreactive fibers in WT mice (C) almost undetectable in IR-hCB1^-/-^ animals (D). Enlarged images (60x), of CG3 area showing CB1R staining (green) in throughout the neuronal processes from WT (E) and transgenic mice (F). Nuclear staining of DNA with DAPI (blue) revealed that, contrary to the WT, in IR-hCB1^-/-^ the CB1-IR diffuses into cytoplasm and almost lacks in neuronal membranes and fibers.

To validate the model, the activity of the reporter gene *LacZ* was tested in IRh-CB1^-/-^ mice treated or untreated with Dox. As shown in Fig. 2, β-galactosidase activity was present in all the brain areas where *LacZ* expression was driven by the CaMKII promoter, whereas a strong reduction in the activity was detected in Dox-treated mice. Fig. 3 shows the mRNA expression levels obtained after real time-PCR in different brain areas. Untreated IRh-CB1^-/-^ mice presented levels of CB1 transgene expression of 21% in the caudatum, 49% in the hippocampus and 72% in the prefrontal cortex in WT animals, whilst 15 days of Dox strongly down regulated its expression in these areas. The prefrontal cortex showed the highest expression level with respect to all other areas. In our model, RNA transgene expression of CB1R revealed levels ranging from 21% to 72% in different brain areas. mRNA expression in cerebellum was absent in the IRh-CB1^-/-^ line under Dox as well as without the antibiotic treatment in comparison to WT mice (panel D), in agreement with the Lac Z data (data not shown). In IRh-CB1^-/-^ immunohistochemistry experiments using the antibody for the last 15 amino acids of the rat CB1R revealed a low signal (Fig. 4, A–D), which was shown in detail (green) to be diffused at cytoplasmic level by nuclear staining of DNA with DAPI (blue), clearly contrasting with that obtained in WT mice (Fig. 4, E–F) were the signal follows the fibers and surrounds neuronal cells. Furthermore, the GTPγS binding assays carried out in the some brain areas from IR- CB1^-/-^ resulted in only 6% (prefrontal cortex) or 13% (striatum and hippocampus) increases of bound GTPγS above baseline after stimulation with the CB1R agonist WIN 55,212-2 (10 µM) (data not shown).

Even though the original construct was fully sequenced before the production of transgenic mice, we decided to sequence the CB1 transgene in the founder animal and in the F1 progeny.

Sequencing of the full CB1 transgene RNA revealed an accidental single nucleotide mutation with a consequent substitution of a methionine with a threonine at position 36 of the receptor protein (M36Th) in the extracellular N-terminal domain. Computational analysis by SIFT (http://sift.jcvi.org/, [44]) revealed that such a substitution would not compromise CB1R function. However, some modifications in protein folding cannot be ruled out.

### Behavioral Experiments

#### Spontaneous motor activity

All male mice were tested for their spontaneous motor activity expressed either as basal horizontal (distance in cm) or vertical activity (number of rearing episodes) analyzed over a 90-minute period in a Digiscan Animal Activity Analyzer.

**Figure 5:**
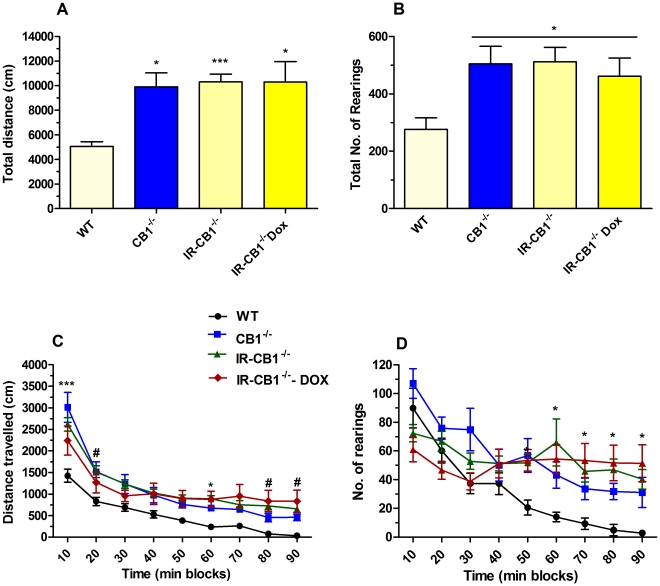
Motor activity in IRh-CB1^-/-^, CB1^-/-^ and WT mice. In panel A and C, the total distance (cm) traveled in 90 min and related time course, respectively, are depicted. The vertical activity expressed as total number of rearing episodes and the time course are illustrated in panel B and D, respectively. All data represent the mean±S.E.M. of WT (n = 9), CB1^-/-^ (n = 8), IRh-CB1^-/-^ (n = 22) and IRh-CB1^-/-^+ Dox (n = 11) mice. For the rearing behavior for some mice the data have been lost (not recorded by the apparatus) and thus the number of mice for the two latter groups is 18 and 9, respectively. ***P<0.001 and *P<0.05 vs WT mice (Dunn’s Multiple Comparison test for total distance, and Newman-Keuls Multiple Comparison test for all the others).

As shown in Fig. 5 (panel A) the IRh-CB1-/-, either treated or untreated with Dox (12 days at the dose of 50 µg/ml), and CB1-/- mice presented significantly higher traveled distances, expressed as total distance, compared with WT animals (P<0.001, Kruskall-Wallis test). Two-way ANOVA with repeated measures showed the significant main effects (Fig. 5, panel C) of genotype (F_3, 368_] = 5.908, P = 0.002), and time course (F_8, 368_ = 94.40, P<0.0001), as well a significant time course x genotype interaction (F_24, 368_ = 2.414, P = 0.0003). In panel B and D the total number of rearing episodes and their time course, respectively, are shown. As for the distance travelled, a statistically significant increase was observed in all transgenic mice compared to WT animals (F_3, 40_ = 3.480, P = 0.025). As for distance, significant effect of time (P<0.0001), genotype (P = 0.03) and genotype x time interaction (P<0.0001) were observed for vertical movements. A significant increase in the time spent at the center of the activity cages (first 10 minutes test) was observed only for the CB1^-/-^ group but not for the WT and the IRh-CB1^-/-^ groups. On the contrary no differences were detected for the time spent at the margins for all groups (data not shown).

#### Pre-pulse inhibition (PPI)

**Figure 6:**
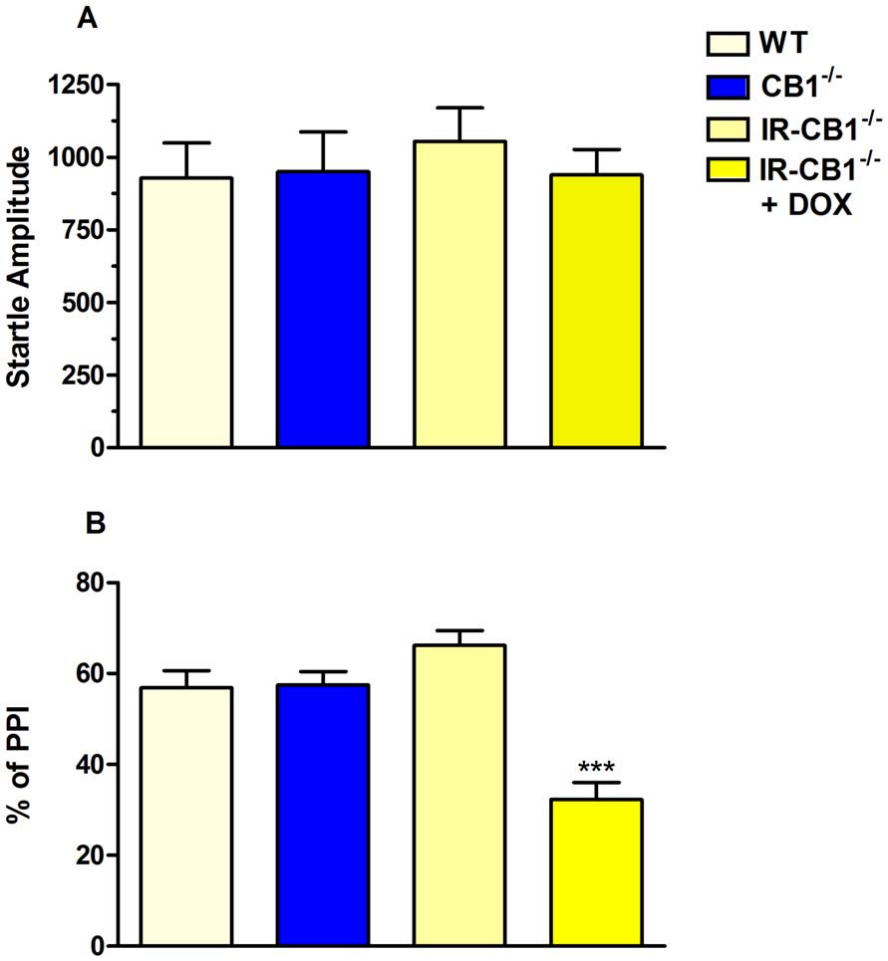
Startle response and percentage of PPI in IR-hCB1^-/-^, CB1^-/-^ and WT mice: effect of Dox treatment. The startle amplitude (panel A) and % of PPI (panel B) are the mean±S.E.M. of WT (n = 13), CB1^-/-^ (n = 13), IR-hCB1^-/-^ (n = 15) and IR-hCB1^-/-^ Dox (n = 14) mice. For PPI the data are expressed as the average PPI response over the 3 prepulse intensities (see supplemental files for the figure illustrating PPI by prepulse intensity). ***P<0.0001 IR-hCB1^-/-^ Dox vs all other groups (Newman-Keuls Multiple Comparison test).

The analysis of the startle amplitude is shown in Fig. 6 (panel A). All mice independently from genotype did not show differences (Fig. 6, panel A) in acoustic startle response amplitude (P = 0.8486). Moreover, when analyzing PPI response in each experimental group at the 3 different prepulse intensities, we did not find significant differences in the response to prepulse intensity within each group. Thus, the data have been expressed as the average PPI response over the 3 prepulse intensities. On the other hand, the one-way ANOVA for the different genotypes showed a significantly decrease in PPI (F_3, 51_ = 20.18, P<0.0001) only in IRh-CB1^-/-^ mice under Dox compared with all other groups. The two-way ANOVA with repeated measures revealed the significant main effect of genotype (F_3, 102_ = 19.73, P<0.001), whereas neither pre-pulse intensity (F_2, 102_ = 0.8939, NS), nor genotype x pre-pulse intensity interaction (F_6, 102_ = 1.127, NS) were significantly affected (see Figure S1 in supporting information data). Therefore, the reduction of PPI observed in IRh-CB1^-/-^+Dox mice was independent of the pre-pulse intensity tested.

**Figure 7:**
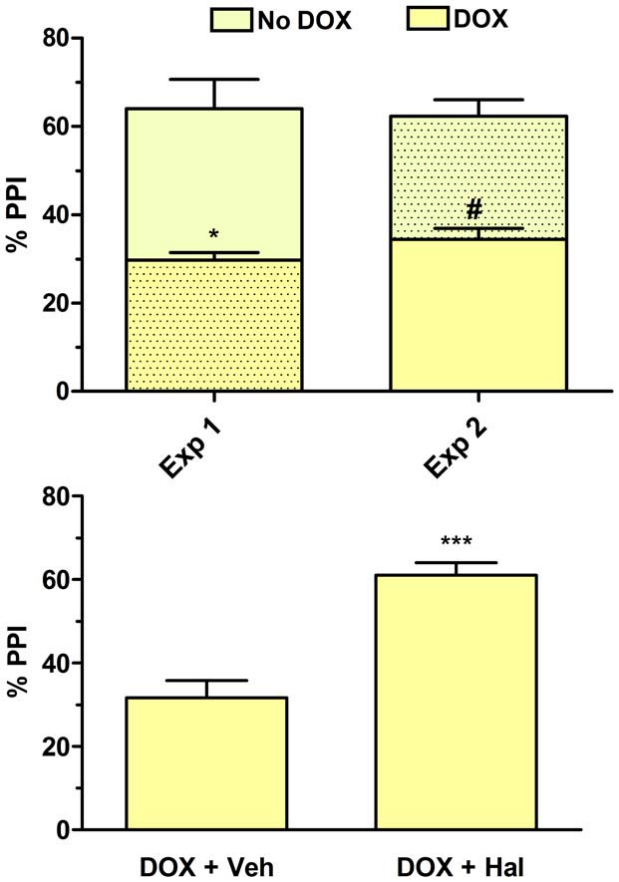
Percentage of PPI in IR-hCB1^-/-^ mice under different conditions. All values represent the mean±S.E.M. Panel A: reversibility of Dox-induced disruption of PPI. Two groups of IR-hCB1^-/-^ mice have been randomly assigned to vehicle group (dotted color, n = 6) or Dox group (plain color, n = 7) and then tested for PPI (Exp. 1). Dox administration lasted 12 days, and extra 12 days served for the washout, after which the schedule was repeated by switching the treatments (i.e. Dox given to previous No-Dox treated mice, and vice versa) (Exp. 2). Panel B: Dox-induced disruption of PPI is antagonized by haloperidol (1 mg/kg i.p., 30 min before PPI session).

All parameters for startle and PPI experiments (startle amplitude P120, first 2 blocks and response to the 3 different prepulse intensities, no pulse and peak time) have been included in the Supporting Information data (Table S1). The reversibility of the Dox effect on PPI values is shown in Fig. 7 (panel A). As previously described, when CB1R expression was knocked down by Dox, a clear significant disruption of PPI was observed after a washout (12 days) from Dox for the treated animals, the two groups of mice were switched in treatment assignment and the PPI values were measured once more. As shown in the panel A the mice previously untreated with Dox, switching off CB1Rexpression induced a significant disruption in PPI; on the contrary, the mice previously in disruption showed, after washing out of Dox, a complete recovery in their sensory-motor gating responses (two-way ANOVA: treatment effect F_1,19_ = 26.42, P<0.0001). To verify the possible involvement of the dopaminergic system in the disrupted PPI, a further experiment was performed. One more group of Dox-treated IRhCB1^-/-^ mice were injected with the dopamine D_2_ receptor antagonist haloperidol (1 mg/kg i.p. 30 min. prior testing). Antagonizing dopamine D_2_ receptors significantly reverted (P<0.001) the PPI disruption induced by knocking down CB1R, with values almost returning to baseline (Fig. 7, panel B). Interestingly, heterozygous IR-hCB1^+/–^, Dox treated and untreated, were also tested for their PPI response. These mice, irrespective of Dox treatment, did not differ in their response from that of the untreated homozygous mice (data not shown).

## Discussion

The present study describes the generation of CB1R conditional and reversible knockdown hypomorphic (IRh-CB1^-/-^) mice with some interesting endophenotypes through the combination of the Tet-off system with the classic knock-out system. Such a strategy provides several advantages over the conventional conditional gene-targeted or chimeric mice. The tet-off system enables the study of mouse *cnr1* gene function in a regulated manner. It allows, through the use of the specific forebrain CaMKIIα promoter for driving tTA expression, gene expression in a reversible and spatio-temporal way otherwise impossible to achieve by the conventional irreversible Cre-mediated conditional knockout system [34]–[35]. Whereas our model works very well in terms of modulating gene expression of the CB1R, some limitations are present in the protein expression (low protein synthesis). The immunohistochemistry experiments show an absence of the receptor from the axonal membrane and suggest an exclusive intracellular localization [45]–[46] and/or a constitutive internalization [47]. Such unexpected results might be reasonably ascribed to the point mutation M36Thr obtained accidentally that affects the extracellular receptor tail. As mentioned above, IRh-CB1^-/-^ mice exhibit altered endophenotypes that include increased locomotor activity and deficits in pre-pulse inhibition of acoustic startle response. These IRh-CB1-/- mice present hyperlocomotion similarly to the classic CB1^-/-^ mice, and it may be ascribed to the unbalanced activity of the neural population of brain circuits involved in controlling motor behavior. Indeed both classical KO mice and hypomorphic mice lack of CB1Rs in different parts of the vertebrate motor system from networks responsible for the execution of movement to planning centers in the basal ganglia and cortex [48]. Such increased spontaneous activity in these mice is partially in contrast with the data reported by Marsicano and coworkers [49] where no difference in locomotor activity between their CB1^-/-^ and WT mice was noted. Such lack of difference seen in locomotor activity might be explained by the short observation time chosen (30 min) and that in this short period the WT did not fully habituate. However, our WT mice were less active than the transgenic ones from the beginning of the experiment, and in particular in the first 30 min, where a significant difference in the motor activity (P<0.05 WT vs all transgenic groups, Newman-Keuls Multiple Comparison Test) could be observed, with the habituation in transgenic mice slower than WT. Furthermore, they carried out their behavioral test during the dark phase and this could obscure differences in activity due to a ceiling effect, being the mice more active nocturnally [21]. Since CB1R are mainly located on GABAergic neurons Monory and coworkers [50], have dissected the behavioral responses to THC in conditional knockout mouse lines lacking CB1 in different neuronal subpopulations (principal brain neurons, GABAergic neurons, cortical glutamatergic neurons, those expressing the dopamine receptor D1, respectively). In their paper it has been suggested: I, that the CB1Rs mediate many activities by different neuronal circuits, which can be dissected by genetic approaches; that those on GABAergic interneurons do not appear to mediate the effects of THC; II, that cortical glutamatergic neurons expressing CB1R mediate a large portion of hypolocomotor effects of cannabinoids and III, that the simultaneous activation of CB1Rs located on striatal neurons and glutamatergic neocortical neurons is likely to be necessary to exert the cataleptic effect of THC [50]. Furthermore, the other complicated interactions between serotonergic and endocannabinoid systems [51] as well as the finding that GABAergic neurons, specifically the β2 subunit, possess a binding site for the endocannabinoid 2AG [52] may differently interfere in locomotor responses in such mice. Moreover the involvement of the HPA axis activation could not been ruled out in the behavioral responses observed in our mice [21], [53], in particular for the different extent of thygmotaxis showed by classical in comparison to WT and IRh-CB1^-/-^ mice. The lack of difference observed in the locomotion behavior between constitutive CB1^-/-^ and IRh-CB1^-/-^ mice, either treated or untreated with Dox, is not surprising, since it has been recently shown that male mice lacking CB1 receptors display decreased voluntary running compared with their wild-type littermates, when housed with a running wheel for several weeks. Moreover, when the daily patterns of running behavior in mice CB1 KO offered running wheels were compared to those of locomotor activity in the ones housed without running wheels) only the former, but not the latter, behavior is under the control of CB1 receptors not being affected by the absence of CB1 receptors [54].

The present study shows for the first time that “acutely” knocking down the CB1R expression in the IRh-CB1-/- mouse treated with Dox is sufficient to unmask an important endophenotype involved in schizophrenia, i.e. the disruption of PPI. Furthermore, the same response cannot be seen in classical CB1^-/-^, with these mice not showing any alteration in their sensory-motor gating responses. This dichotomy might be explained by the presence of compensatory mechanisms occurring in the constitutive CB1^-/-^ mice and by the particular neuroanatomical circuitry mediating the PPI response [55]. Indeed, PPI can be regulated, and even eliminated, by subtle pharmacological manipulations at the most rostral tip of the forebrain: i.e., PPI is mediated via the pons and can be regulated by the forebrain [55]. In our case only “acutely” switching off the, although not fully expressed, receptor in the forebrain is sufficient to affect PPI, a behavioral test that together with motor activity is frequently used for validating animal models of schizophrenia.

It is also noteworthy that rimonabant, the inverse agonist for CB1 receptors, even at doses that usually antagonize the effects of CB1 stimulation, is unable to induce PPI in either mice or rats [1], although it is able to inhibit the disruption of PPI induced by the dopamine D2 agonist apomorphine [27]. Moreover, it must be underlined that the Dox-induced PPI disruption was completely reverted when the mice underwent Dox withdrawal, i.e. when the CB1R expression was restored. Vice versa it was again observed in those IRh-CB1^-/-^ mice previously treated with vehicle alone. Such result is peculiar of this model since the knocking down of the CB1R is not permanent as it is for the corresponding CRE-Lox mice [39]. Thus, the benefit of our model is represented by its reversibility. In this it might be more interesting to investigate on the related modifications and interactions in different brain areas that can be obtained during such gene manipulation. One interesting interaction is obviously with the dopaminergic system and, in Dox^+^ IRh-CB1^-/-^ mice; the administration of 1 mg/kg haloperidol completely antagonized the impairment of sensorimotor gating, consistent with the involvement of that neurotransmitter system. Future experiments for both behavioral and gene expression alterations in such crucial neuronal systems, as well as for the other ones (GABAergic, Glutamatergic and so on) will be needed and in particular for the glutamatergic system that is supposedly being disregulated (hypofunction) in schizophrenia [56]–[57] However, since the expression of the CB1R in hypomorphic IRh-CB1^-/-^ mice is very low, the PPI disruption observed might also be attributed to CB1R intracellular activation [47], [58] and thus it deserves more experiments to investigate other intracellular pathways linked to CB1 activation (increase in MAP kinase phosphorylation and decrease in cAMP production). The observed M36Thr substitution at the N-tail could indeed prevent their complete localization at the plasma membrane.

Contrary to the other members of the GPCRs, the CB1R has an exceptionally long extracellular N-terminal domain (N-tail) of 116 amino acids but without the typical cleavable signal sequence. It has been suggested that the long N-tail affects the biosynthesis of the receptor and its insertion into the endoplasmic reticulum (ER) membrane [59]. Indeed, when the long N-tail is not efficiently translocated across the ER membrane, the CB1R undergoes rapid degradation by proteasomes, leading to its low expression at the plasma membrane; on the contrary, adding a signal peptide at the N-terminus of CB1R or shortening the long N-tail increases stability and cell surface expression [59]. The M36Thr substitution could then enhance the degradation rate by proteasomes or affect in some way receptor binding to the CP-55,940 and account for the low protein expression in the IRh-CB1^-/-^ mice. Alternatively, since no cleavable signal sequence is present at the N terminus, the point mutation in the IRh-CB1^-/-^ mice might change the folding behavior of the N-terminal domain, an important factor in the topogenesis of signal-anchor proteins [60]. Usually, a GPCR resides at the cell surface and, following activation, undergoes phosphorylation, desensitization, internalization and finally either degradation or recycling [61]. Moreover, while it is internalized, the receptor may also contribute to activate signaling cascades such as those first identified for the β_2_-adrenergic receptor and more recently for the angiotensin II 1A receptor [58], [62]–[63]. Immunocytochemical staining both in cell cultures and in neurons has demonstrated that CB1Rs constitutively internalize in their native state [64] although their trafficking resulted to be highly complex [65]. It has been recently described by Rozenfeld and Devi [58] that transfected and tagged CB1Rs preferably localize at the plasma membrane, whereas endogenous receptors are intracellular.

The lipophilic properties of the ligand are crucial for its ability in crossing the membrane and activate intracellular receptors [46]–[47]. It is likely that GPCRs which bind a lipophilic ligand may be activated at intracellular compartments. Among GPCRs, the CB1R binds and is activated by the lipid derivatives such as anandamide and 2-AG [58]. These endogenous cannabinoids are extremely lipophilic and easily diffuse through the plasma membranes [59]. Moreover, endocannabinoids are actively uptaken into the intracellular compartment although no specific transporters are yet identified [46] and stimulate intracellular CB1R [66]. In our case, it can be also hypothesized that the point mutation in the receptor preferentially shifts the equilibrium to the internalized form.

The generation of a full IR-CB1^-/-^ mice without a point mutation is in progress and will be utilized to validate that the “acute” knockdown of the receptor after Dox treatment accounts for the PPI disruption observed in the present study. However, the lack of alteration of PPI in canonical CB1^-/-^ mice must be stressed, although a PPI disruption in other “cre-lox” conditional CB1^-/-^ mice [34]–[35] cannot be ruled out, no such kinds of experiments have been performed to date.

Recently, the deletion of CB2r in CB2^-/-^ mice has been reported to induce schizophrenia-related behaviors, including PPI disruption and reduced motor activity [10]. In this paper, the distinctive profiles between patients with bipolar disorder and schizophrenia were used in support of their CB2^-/-^ mice as a model of schizophrenia. They suggested that mutant mice with motor hyperactivity and reduced PPI, similar to patients [67]–[68], were more closely related to animal models of bipolar disorder than schizophrenia. Since our hypomorphic IRh-CB1^-/-^ mice showed spontaneous increased motor activity and the “acute” knockdown of CB1R expression induced by Dox impaired PPI, such a suggestion deserves further studies and a more complete behavioral and biochemical characterization using the still in-progress full IR-CB1^-/-^ mice.

Conclusions: In the classic CB1^-/-^ mice, there were no changes in PPI, but in our IRh-CB1-/- mice under Dox treatment, PPI was disrupted, which was easily reversible after withdrawing the Dox or after Haloperidol treatment. We describe for the first time the clear involvement of CB1R in PPI disruption, which might shed light on the contradictory data reported up to now. Although hypomorphic due to a *de novo* point mutation, our IRh-CB1-/- mice might represent a powerful tool for studying the physiological and pathological roles of the cannabinoid system given its reversibility in gene expression, and better investigate the complex interaction between the endocannabinoids and the other neurotransmitters. However, given that most of the disrupted genes are involved in neuronal plasticity, glutamatergic or dopaminergic function and synaptogenesis [2], [69] and multiple susceptibility genes act synergistically, as well as in conjunction with epigenetic processes and early-life environmental adverse effects, sophisticated strategies are needed for the development of novel schizophrenia models. Recently it has been proposed that rather than focusing on a mutation or an altered function of only a single gene, a better strategy is produce double or triple genetically modified mice to explore the interacting effect between different genes [2].

## Supporting Information

Figure S1 **Percent of PPI in IR-hCB1-/-, CB1-/- and WT mice at different prepulse intensities: effect of Dox treatment.** The % of PPI at the different prepulse intensities are the mean±S.E.M. of WT (n = 13), CB1-/- (n = 13), IR-hCB1-/- (n = 15) and IR-hCB1-/- Dox (n = 14) mice. The two-way ANOVA with repeated measures revealed the significant main effect of genotype (F3,102 = 19.73, P<0.001), whereas neither pre-pulse intensity (F 2,102 = 0.8939, NS), nor genotype x pre-pulse intensity interaction (F6,102 = 1.127, NS) were significantly affected.
(TIF)

Table S1 **Values represent mean±S.E.M. for each parameter (for further details, see text).** Mean SA, mean startle amplitude for the whole trial sequence; First block, mean startle amplitude for the first half of the session; Second block, mean startle amplitude for the second half of the session; PP3, PP6 and PP12, mean prepulse levels at the different intensities; No pulse, mean no pulse, expressed as percent of whole trial sequences; Peak t., mean of the latency to the peak of startle for the whole trial sequence.
(DOCX)
