# Supplementary figures and images for: Reversible Disruption of Pre-Pulse Inhibition in Hypomorphic-Inducible and Reversible CB1-/- Mice
Source: PLoS One. 2012 Apr 27;7(4):e35013. doi: 10.1371/journal.pone.0035013 (PMC3338749; doi:10.1371/journal.pone.0035013)

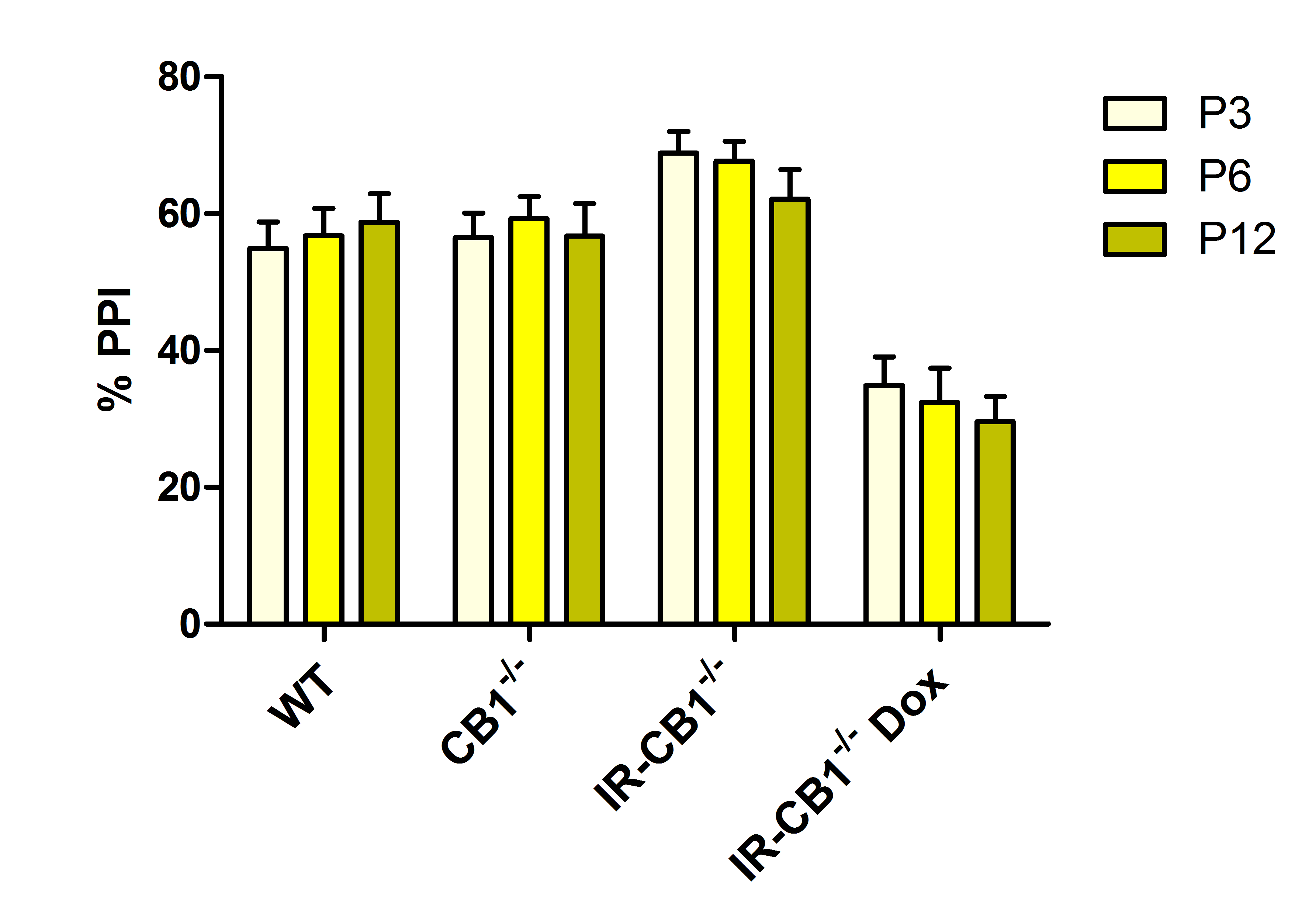

Supplement: Figure S1 — Percent of PPI in IR-hCB1-/-, CB1-/- and WT mice at different prepulse intensities: effect of Dox treatment. The % of PPI at the different prepulse intensities are the mean±S.E.M. of WT (n = 13), CB1-/- (n = 13), IR-hCB1-/- (n = 15) and IR-hCB1-/- Dox (n = 14) mice. The two-way ANOVA with repeated measures revealed the significant main effect of genotype (F3,102 = 19.73, P<0.001), whereas neither pre-pulse intensity (F 2,102 = 0.8939, NS), nor genotype x pre-pulse intensity interaction (F6,102 = 1.127, NS) were significantly affected. (TIF) [file pone.0035013.s001.tif]
