# Supplementary material for: Reversible Disruption of Pre-Pulse Inhibition in Hypomorphic-Inducible and Reversible CB1-/- Mice
Source: PLoS One. 2012 Apr 27;7(4):e35013. doi: 10.1371/journal.pone.0035013 (PMC3338749; doi:10.1371/journal.pone.0035013)
Supplement: Table S1 — Values represent mean±S.E.M. for each parameter (for further details, see text). Mean SA, mean startle amplitude for the whole trial sequence; First block, mean startle amplitude for the first half of the session; Second block, mean startle amplitude for the second half of the session; PP3, PP6 and PP12, mean prepulse levels at the different intensities; No pulse, mean no pulse, expressed as percent of whole trial sequences; Peak t., mean of the latency to the peak of startle for the whole trial sequence. (DOCX) [file pone.0035013.s002.docx]

Table S1 – Mean startle amplitudes and relative parameters in WT, CB1^-/-^ and (**IRh-CB1**^-/-^)

Genotype ST. AMPL Block Prepulse level

(No.) P120 1st 2nd PP3 PP6 PP12 NO PULSE PEAK T.

WT (13) 927.8±120.9 995.1±113.7 852.0±133.2 54.9±3.9 56.8±4.0 58.8±4.1 81.6±3.7 49.9±0.8

CB1^-/-^(13) 949.3±137.1 1032.4±155.1 855.7±124.7 56.5±3.6 59.3±3.2 56.7± 4.8 84.8±2.6 51.6±1.2

**IRh-CB1**^-/-^ 1101.3±115.5 1094.1±112.1 1109.4±121.9 68.8±3.2 67.8±2.9 62.1±4.3 86.7±2.8 51.6±0.6

(15)

**IRh-CB1**^-/-^ 1013.3± 95.3 1045.4±118.7 977.2±106.6 34.9±4.2 32.4±5.0 29.6±3.7 87.7±2.1 50.8 ±0.5

+Dox (14)
